# Design of Laboratory Stand for Displacement Measurement of IPMC Actuators

**DOI:** 10.3390/s23031271

**Published:** 2023-01-22

**Authors:** Karina Koślik, Paweł Kowol, Rafał Brociek, Agata Wajda, Grazia Lo Sciuto

**Affiliations:** 1 Department of Mechatronics, Silesian University of Technology, Akademicka 2A, 44-100 Gliwice, Poland; 2 Department of Mathematics Applications and Methods for Artificial Intelligence, Faculty of Applied Mathematics, Silesian University of Technology, 44-100 Gliwice, Poland; 3 Institute of Energy and Fuel Processing Technology, Zamkowa 1, 41-803 Zabrze, Poland; 4 Department of Electrical, Electronics and Informatics Engineering, University of Catania, Viale Andrea Doria, 6, 95125 Catania, Italy

**Keywords:** ionic electroactive polymers, displacement measurement, thermal camera, electrical characterization

## Abstract

The polymer technology based on Electroactive polymers and metal composite ionic polymer has great potential and advantages in many engineering fields. In this paper, a laboratory stand for testing Ionic polymer–metal composites (IPMC) is presented. The laboratory station includes a power supply system and a measuring system for the displacement of IPMC composites. Tests and measurements are carried out using a laser transducer and a camera equipped with image analysis software to determine the IPMC strips displacement. The experimental investigation of IPMCs under different voltage supplies and waveforms, environmental working humidity conditions, temperature, and loading conditions has proved the significant influence of geometric dimension and the effect of increased stress on the displacement value. For materials powered by a higher voltage value, an increased deflection value was noted. In case of displacement, longer is the sample, higher is the displacement value. The length of the sample under load, affects adversely its performance, resulting in an increase in the load on the sample. For samples of a thick size, a more stable movement with and without load can be noticed.

## 1. Introduction

The polymer technology has several advantages in many engineering fields related to mechanical properties, and important characteristics of membrane’s transparency and low material thicknesses. The polymer materials are used also in civil structures and building to manage specific humidity and ventilation and to prevent interstitial condensation in multi-layer membrane structures, and in medicine as biomimetic sensors, bio-engineered robots, actuators and artificial muscles [1,2,3,4,5,6,7]. Intelligent Smart materials as Shape Memory Alloy, dielectric elastomers, Ionic electroactive polymer(IEAP), gel–metal composites and piezoelectric are included in the bionic robots [8]. The parameters of these materials can be modified by the control of the appropriate external stimuli or voltages; simulating the muscle contraction in artificial muscles [9,10].

The electroactive polymers are capable of converting electrical energy into mechanical energy changing their size or shape. Under an electric input powered by external voltage sources, they can reveal high elongation, high energy density, and large deformation [11,12,13]. In particular, the Ionic polymer–metal composite (IPMC) is an electroactive and thin membrane coated with a noble metal, usually PMCs are composed of an ionic polymer such as Nafion or Flemion, chemically plated or physically coated with platinum or gold conductors [10,14,15,16,17]. On the other hand, the action of IPMC materials is caused by the movement of ions inside the polymer at low voltages [9,18,19]. By applying a low voltage across the two membranes, the internal hydrated cations migrate towards the cathode. This migration creates an osmotic pressure gradient throughout the membrane. As a result, deformation or bending occurs toward the anode side. Moreover IPMCs have a nonlinear behavior in many applications. The non-feedback method for eliminating the “back relaxation” effect of non-patterned IPMCs by using a relatively high-frequency disturbance is proved in [20]. The doping of polyethylene oxide PEO reinforced electromechanical performances and restrained displacement attenuation of the resultant IPMC [21]. The IPMC-Li samples are used as actuators because of their rapid actuation movement, large displacement and absence of apparent back-relaxation [22]. The Ionic Polymer Metal Composites produce natural actuation performance and can reversibly deform to various external stimuli as artificial muscles or actuators [23,24,25,26]. Authors of the study [27] conducted research on the mechanoelectrical transduction characteristics of this material. The results showed that the enrichment of IPCM with a Nafion nanofiber mat improves its mechanoelectrical transduction properties. Fluorinated acrylic copolymer membrane, Sulfonated polymers, blended ion-exchange membrane of Nafion and poly(vinyl alcohol-co-ethylene) [P(VA-co-E)] have been widely used in IPMC actuators. The IPMCs are characterized by Fourier transform infrared spectroscopy, differential scanning calorimetry, cross-sectional morphology and in term of electromechanical performances [28,29]. The IPMCs applications can be found in [21,30,31,32] since they are the most promising development materials. Although many models are useful for studying the capabilities of electroactive polymers and characterization bench [30,31] this article focuses on design of experimental laboratory stand and characterization of IPMCs materials.

**Figure 1:**
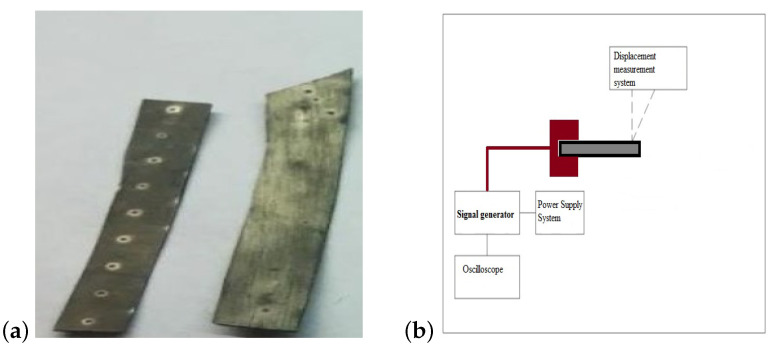
Tested electroactive polymers (**a**) and the test stand (**b**).

The aim of this research is to design and build a laboratory stand for testing ionic electroactive polymers. The lab station includes a power supply system and a measuring system for the generated force and the displacement of IPMC composites. Displacement IPMC strips tests and measurements are carried out to determine their peculiar characteristics and important applications. The measurement system is presented in Figure 1b. The oscilloscope is used for measurement of current requested by the IPMC strips. The material displacement measurement system is implemented in two ways using firstly a laser transducer and then a camera with image analysis software for testing ionic electroactive polymers. The characteristics of the displacement are determined during the movement of the strips powered by a battery and an external laboratory power source. The electroactive samples have been investigated under external load. Wet ionic electroactive polymer materials operated under conditions in higher humidity and displacement rate. The measurements for each of the samples were combined to analyze the effect of increased load on the displacement value.

The paper is structured as follows: in Section 2, the IPMC samples are described; in Section 3 and Section 4 the experimental set up and the measurements are reported, respectively. Conclusions are presented in Section 5.

## 2. Materials

### 2.1. Electroactive IPMC

**Figure 2:**
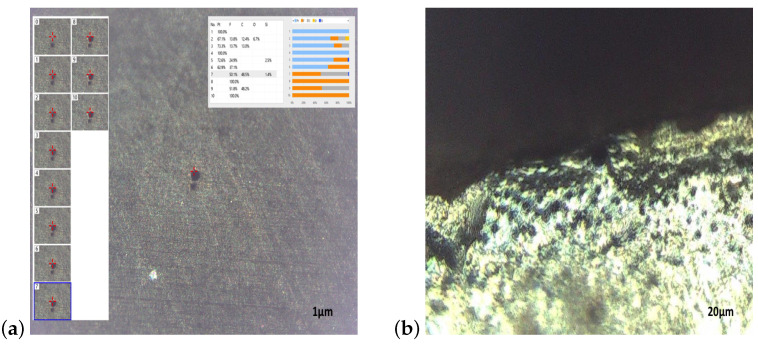
The region of the tested electroactive polymer IPMC based on Nafion/Pt under the Keyence Digital Microscope was examined. The analysis of the mass spectra of the IPMC has provided and has localized the spatial distribution of specific molecules and compositional in % of carbon, silicon powders, oxygen, platinum and fluorine (1 μm) (**a**) and Surface morphology of IPMC material (20 μm) (**b**).

The Electroactive IPMCs have an ionic membrane covered on both sides with a layer of precious metal—usually platinum or gold. The membrane is most often made of nafion. It is a synthetic material consisting of a copolymer of tetrafluoroethene (TFE) and 2-perfluoro(alkyl)ethyl vinyl ethers with acid sulfone residue. This material is most often in the form of a thin foil and it is chemically and thermally resistant. Moreover, it has a shape memory. The morphological characteristics of IPMCs are based on metal composite components. The realized IPMC consists of Nafion ionic polymer membrane with metal electrodes of platinum film, as shown in Figure 2a. The tested samples of electroactive material are strips of ionic electroactive polymers as reported in Figure 1a. They have different geometrical properties, as shown in Table 1, and have been fabricated using the electroless plating method. The IPMCs are fabricated by depositing a Pt metal electrode layer onto the surface of the nafion films through electroless plating. The electroless plating process allows the easy autocatalytic deposition and production of composite film and coatings. The electroless plating utilizes the reaction of metastable redox pairs (e.g., $H_{2}PtCl_{6}$, $RuCl_{3}$), formed by dissolved reducing agents and metal ions or complex compound for achieving the formation of metallization [32,33,34,35].

### 2.2. Fabrication of IPMCs

The IPMCs were fabricated by the hot-pressed method and the Nafion films were located to improve the between the electrode layers and membranes into a hot-press mold at 180 ${}^{\circ }$C [6]. The temperature of the hot press machine was set at 180 °C for 10 min to soften the polymer membrane sufficiently. Then, the pressure of the hot press machine was set at 7 bar, and the temperature was maintained for 30 min. The nafion membrane of IPMCs were roughened by sandblasting under an air pressure of 0.2 MPa. The surfaces of the films were treated by oxygen plasma and deionized water to achieve better adhesion of metal to the surface. After, the nafion film was boiled in a solution of hydrochloric acid and deionized water into an ultrasonic bath for 30 min. The electroless plating process was performed and the nafion films were immersed into a platinum complex solution ($Pt\left(NH_{3}\right)4Cl_{2}\cdot H_{2}O$) and sodium borohydride ($NaBH_{4}$) purchased from Sigma-Aldrich. A Keyence Digital Microscope VHX-7000N was used to observe the morphologies of the IPMCs in different areas after electroless plating Figure 2a. After the electroless plating, a platinum (Pt) metal electrode layer was observed on each IPMC and the presence of Pt on the surface of the IPMCs was confirmed by microscope Figure 2a,b. The KEYENCE Digital Microscope has conducted the elemental analysis referred to the detection of the compositional elements of the target and determination how much of each element there is. The elements within the sample are focused by an ion lens, separated by a mass spectrometer, and measured by a detector. Laser-Induced Breakdown Spectroscopy is a type of inorganic analysis that uses the light emission analysis method. A short-pulse laser with high energy density is projected onto the surface of the sample, converting a small piece of the sample to plasma which is sampled, atomized, and excited. When the part exposed to the laser returns to its ground state, light is emitted. Measuring the wavelength and intensity of this light with a spectrometer or CMOS provides data that allows for the qualitative and quantitative analysis of the elements contained in the sample [36]. The spectra analysis of the tested IPMC was examined under the KEYENCE Digital Microscope exhibiting compound and particles expressed in % of carbon, silicon powders, oxygen, platinum and fluorine. In Figure 2a the maximum contents of molecules and compositional are achieved for platinum and fluorine expressed in 100%. The maximum contents of molecules such as carbon, silicon powders, oxygen are in 48.5%, 2.5% and 6.7%, respectively. The platinum (Pt) content is about 100% in the areas of the analyzed IPMC and the lowest Pt content is 62.9% as shown in Figure 2b. The Pt content and the thickness influence the conductivity of the IPMCs and the surface resistances, measured using a 4 Point Probe Meter Digital, for the analyzed specimen is circa 4.25 Ω/sq. The IPMC with lengths of 30.5 mm and 44 mm reveal at 5 V maximum bending angles of 30${}^{\circ }$ and 45${}^{\circ }$. The bending angles of IPMCs were measured directly using the grid paper and calculating the arc length. After the results have been compared by using the laser displacement sensor ( Keyence LK- 160 G 152 laser transducer) to calculate the distance displacement and applying the Law of Cosine (also called the Cosine Rule) to obtain the bending angle [37].

The electroactive material operates in an environment with increasing humidity. Its stiffness decreases and increases according to its relaxation level. The IPMC materials operate in an environment with progressively lower humidity. The level of relaxation can decrease over time until the strip of material is completely dry. In this case, the movement of the sample can also decline over time until it stops. An ideal humidity level is considered for a balance between movement and relaxation. Different voltages can be applied to electroactive material samples to achieve more complex material strip movements. Comparing the different smart actuators properties, the electroactive actuators show the greatest distortion at a low value of the voltage. They also consume less power than SMART actuators.

## 3. Experimental Tests and Setup

The measurement laboratory stand was designed using the Inventor program and its implementation to test the electroactive polymers have been carried out, as shown in Figure 1b. The experimental test set-up was composed of signal generator, power amplifier, oscilloscope (Tektronix TBS1052C 50 MHz 1 GSa/s) and Laser Displacement Sensor. Displacement and force measurement have been conducted on a variety of strip materials using laser transducers Keyence LK-G152 Laser Displacement Sensor LKG152. The laser displacement sensor has been used to ensure stable, high accuracy measurements range (150 ± 40 mm) and repeatability (0.5 μm). The measurement system has also been powered using the external voltage by the power supply and the voltage recommended by the manufacturer of 9 V battery. The external voltage source and battery have been used to supply the dynamic signal generator, which was connected to the strips of electroactive material. The dynamic signal generator allows to control the pulse width and to reduce or increase the voltage value of electroactive samples and to set the output value of the system voltage. The dynamic signal generator can be powered by 9 V battery or by a regulated external laboratory power supply. The voltage applied to the electroactive material samples was set to 5 V and the current limit was set to 0.4 A.

The current flowing through the electroactive polymers was measured by oscilloscope. The output supply voltage and current were set to 5 V and 0.4 A, respectively. For the electroactive specimen, the maximum values of the current were approximately 0.358 A for power supply from an external voltage source and 0.25 A for battery.

## 4. Results

### 4.1. Displacement Measurement of the Tested Polymers Using the Laser Method

**Figure 3:**
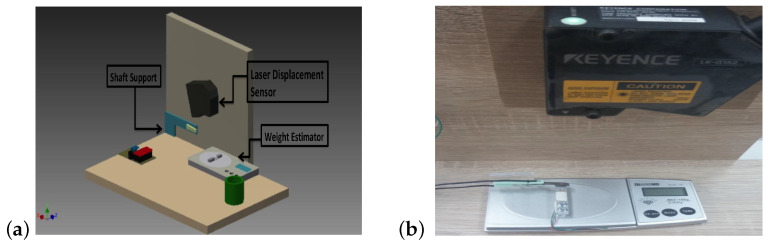
Designed measuring test stand front view (**a**) and measurement conducted with a laser transducer (**b**).

In order to measure the displacement of the electroactive material strips, a Keyence LK-G 152 laser transducer was used. The laser transducer was placed on side of the test stand at a distance of 150 mm from the tested object (Figure 3b). The experiments were carried out for various values of the supply voltages for the material samples. Each measurement is conducted for 6.5 s.

**Figure 4:**
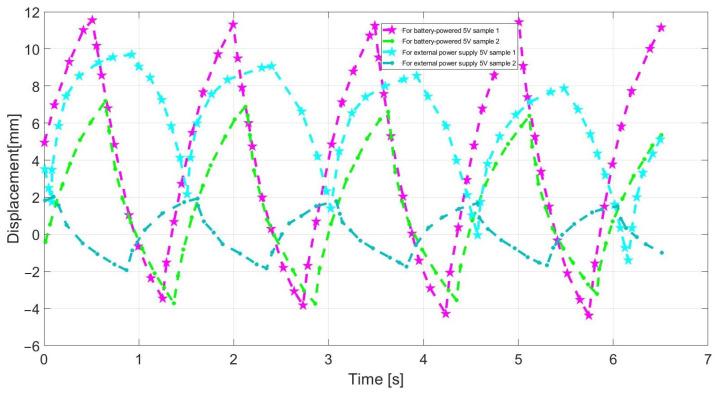
The displacement values for strip samples that had produced good results when powered by an external source and by a battery.

The material deflection was greatest while using a battery power supply with external voltage of 5 V. The maximum deflection value was 15.9 mm for sample 1, which was powered by a battery (Figure 4). For the lowered supply voltage, the smallest displacement was obtained. For the output voltage of 2 V supplying electroactive samples, the smallest displacement value of 3.20 mm was found. The bar of sample 2 has been deflected by around 11 mm from its original position (Figure 4). During the measurement, the strip showed a stable behaviour both during battery operation and when powered by an external voltage source.

The deflection value of a material is affected by external conditions, as the humidity level of the material has the greatest effect during the measurement. As a result, wet strips give more deflection. Therefore, best results were obtained with strips placed in distilled water for 24 h prior to measurement. The voltage values applied to the material also affects the deviation.The result of the lowest deviation was achieved when the supply voltage was lowered. The strips were very wet and there was not current limit, so the best results were obtained with battery power. The measurements were carried out for 6.5 s to determine the relationship between material displacement and supply voltage. Under the applied voltage, the electroactive samples become irregularly shaped, making them difficult to measure their deflection.

### 4.2. Displacement Measurement of Tested Polymers by Image Analysis

**Figure 5:**
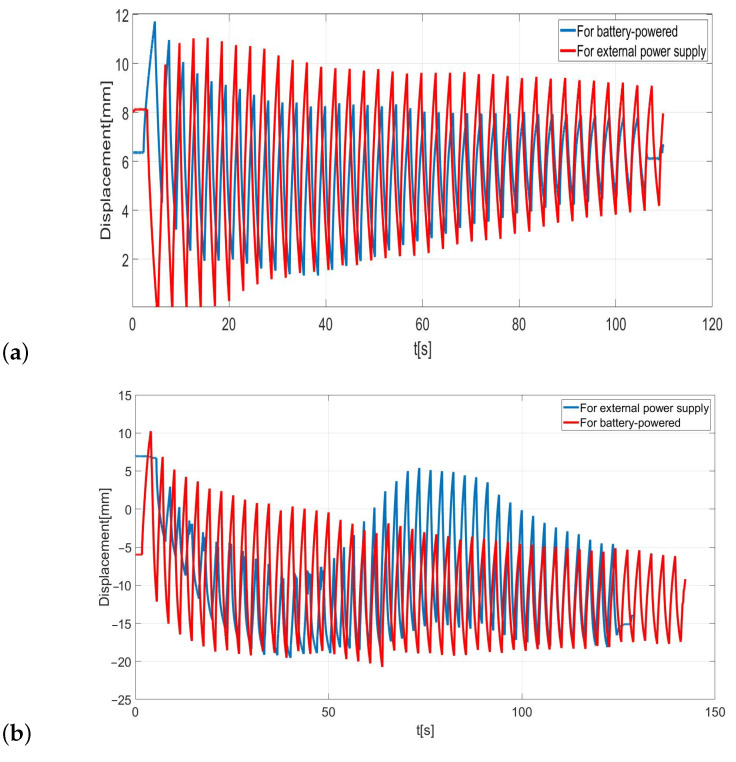
Displacement values determined by computer method for sample 1 (**a**) and for sample 2 (**b**).

The measurements are conducted by image analysis because the displacement of the electroactive material sample is beyond the measurement range. The program used to analyze the image was able to quite accurately determine the displacement of those samples that have generated the largest displacement immediately after the immersion in the water. As a result, the displacement was measured immediately after that the samples were picked from the water in order to generate the greatest displacement. For strip sample 1, using battery power the deflection value was increased. The maximum displacement value was about 11 mm (Figure 5a). During the measurement, the strip operated stably for both battery and an external voltage source supply. The speed of the material strip battery powered was faster than external voltage source.

Due to the dependence obtained by the displacement of the strip sample 2 supplied by an external voltage source, this material could not be controlled, especially in the early stages of its movement. First, the deflection of the sample was very small and gradually increased over time. After about 60 s, the deflection reached its maximum and the strip changes position, and after about 100 s, is stabilized. With the battery-powered sample, the stripe traveled smoothly and gradually decreased over time. The maximum displacement for external power supply was about 22 mm, for the maximum battery power the displacement was about 21 mm (Figure 5b). The measurements based on a computer method of image analysis were burdened by errors resulting from the used camera. Some frames were blurred during the impact of the strip and before the motion was stabilized, so the software program was unable to determine the correct strip position accurately. In addition, the image analysis was very troublesome and time-consuming. The movement of the stripes was smoother when it was supplied by battery, which means that the movement was easier to control for stable results. In addition, the longer is the strip, the more difficult it is to make it move smoothly.

### 4.3. Displacement Measurement of the Tested Polymers under Load

**Figure 6:**
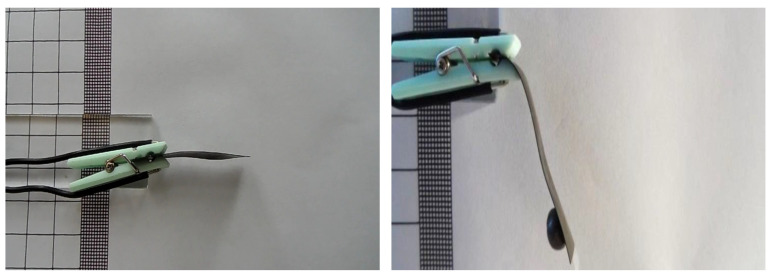
Displacement measurement of the tested polymers under load conducted in laboratory.

The effect and action of electroactive polymer as muscles used to lift 0.2 g, 0.5 g, 1 g weights was analyzed (Figure 6).

**Figure 7:**
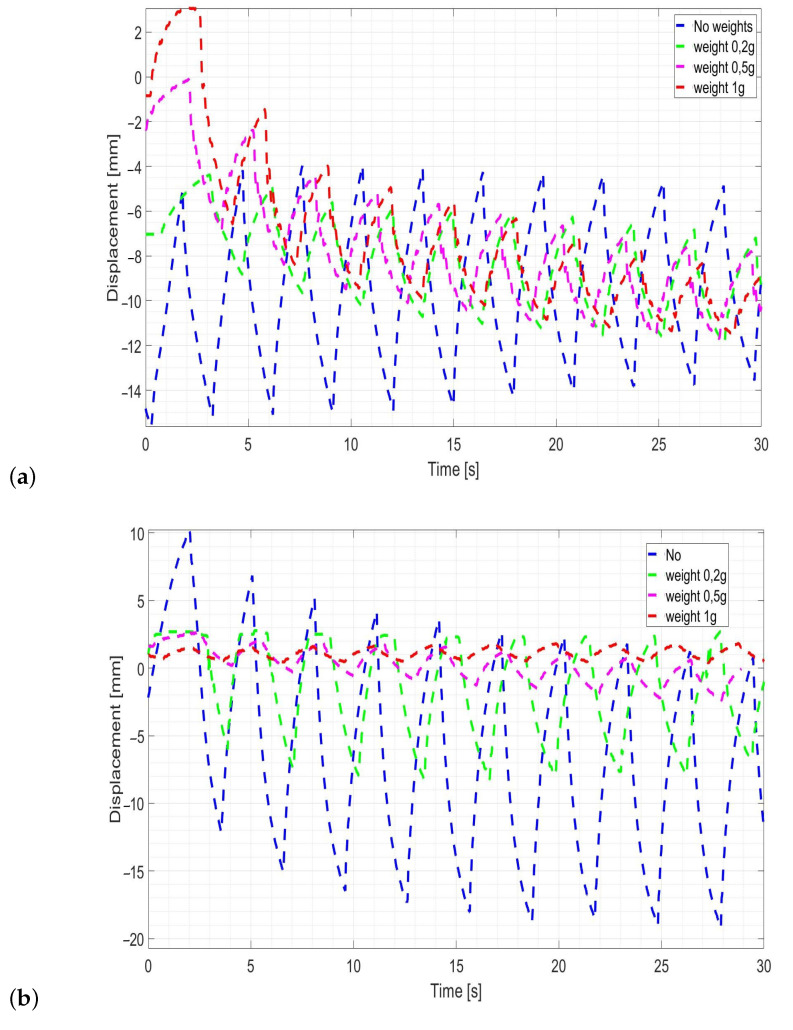
Displacement values of electroactive sample 1 under load (**a**) and electroactive sample 2 (**b**).

A computer method of image analysis was used to compare the movement of each loaded electroactive polymer. The system was powered by a 9 V battery and the voltage applied to the strip was 5 V. Figure 7 shows the displacement measurement results of the obtained test sample with and without load.

The measurement results are satisfactory, for the electroactive polymer sample 1, capable of transferring a weight of 1 g (Figure 7b). This is due to its geometric dimensions—the strip is short and thick (30.5 × 4.4 × 0.3 mm), achieving greater stiffness and the ability to carry small loads. For electroactive material strip loaded with heavier weight, the displacement decreased over time.

For the strip sample 2, very good measurement results were obtained for a load of 0.2 g. The movement was smooth, stable, and the deflection was about half value compared to operating condition without load. The geometrical dimensions play a great role, in this case the strip 2 is greater than the other sample. The electroactive material strip loaded with a weight of 1 g, was tilted approximately of 2 mm. For this material sample, its displacement under the action of external load was the smoothest and most stable compared to the rest of the samples (Figure 7b).

The “strength” of electroactive samples depends on their geometrical dimensions. The samples with the greatest thickness (for example 1, 2 and 7 mm) than sample 1 and 2 used in this experimental tests, with the highest load of 1 g, have reached the greatest displacement in relation to other samples. In this conducted research, the ionic electroactive polymers are not suitable for lifting weights. The strip length has the greatest impact on deflection. If the electroactive polymer works without load, longer is the strip, better is the displacement result obtained. The opposite is true for strips of material operating under load. Moreover, the load moment increases with the dimensions of the electroactive material samples. Additionally, the movement of the loaded strip is very unstable. Its characteristic has an irregular shape with an alternating upward and downward movement. This proves that the strip vibrates during the experiment. For more precise movements and under smaller load, the polymers do not operate so well.

#### Temperature Dependence of Ionic Electroactive Polymers

**Figure 8:**
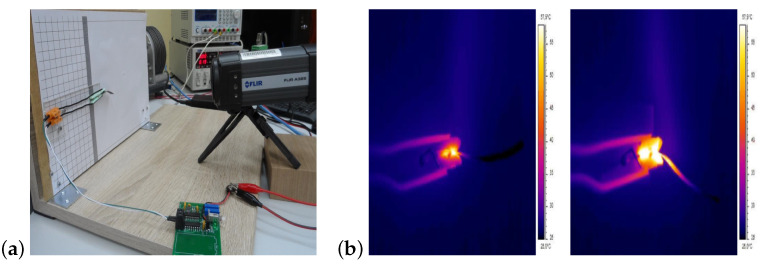
Implemented system with thermal imaging camera (**a**) and measurements carried out by thermal imaging camera for 20 and 60 s on strip (**b**).

During the measurements, the material strips heat up excessively under the influence of the applied tension causing mechanical damage to the material. For this reason, in order to perform measurements the FLIR A325 thermal imaging camera for each of the samples has been adopted in the measuring stand. The thermal camera is used to deliver accurate thermal imaging and repeatable temperature readings. The FLIR A325sc is equipped with an uncooled Vanadium Oxide (VoX) microbolometer detector that produces thermal images of 320 × 240 Pixels. These pixels generate crisp and clear detailed images that are easy to interpret with high precision. The FLIR A325sc will make temperature differences as small as 50 mK clearly visible. In addition to the camera, a computer with an appropriate program for analyzing the image by camera was also required (Figure 8b). However, in all experimental cases the electrodes heat up and transfer heat to the material samples. During the system operation, the electrodes heat up to 50 ${}^{\circ }$C. Measurements using a thermal imaging camera by heating of electroactive polymers for 20 and 60 s have been provided, as shown in Figure 8b.

## 5. Conclusions

Electroactive polymers are considered to be advantageous intelligent materials in traditional electric, pneumatic and hydraulic drives, in medicine for the construction of prostheses, and in robotics for grippers or walking robots. Moreover, they are also used in robots that simulate the human facial expressions and in the construction of snake-like robots. In this research, the designed measuring laboratory station has been implemented to conduct tests with electroactive polymer samples. During these tests, the samples were also powered by an external power supply or laboratory battery. Computer techniques for image analysis have also been used to investigate how the displacement of electroactive samples, powered by a 9V battery and an external laboratory power source, changes. The electroactive samples were investigated under external load. The samples were subjected to a load of 0.2 g, 0.5 g and 1 g. The wet ionic electroactive polymers materials operated in conditions of increased humidity and larger displacement. The measurements for each samples were combined to analyze the effect of the increasing load on the displacement value. The cause of the samples heating up was investigated using a thermal imaging camera. The most important results obtained prove:

- For materials powered by a higher voltage value, an increased deflection value was noted for a power supply with a lower value of the output voltage in the system.
- The geometric dimension also has a significant influence on the behavior of the sample.
- In case of displacement, the longer the sample is, the higher the displacement value is.
- On the other hand, by analyzing a sample under load, the length of the sample affects adversely its performance, resulting in an increase in the load on the sample.
- The movement is less stable and smaller for the lowest weight sample value.
- The thickness of the sample is also of great importance. For samples of thick sized, a more stable movement with and without load can be noticed.

Therefore, the geometric dimensions of the samples can be easily modified to adjust the best parameters as required. In the future works, the analyzed samples of electroactive material could include a detailed analysis of their properties related to environmental air temperature and humidity conditions in which the measurements are carried out. Additionally, to achieve a more accurate displacement measurement, the power supply system and the feedback loop can be designed to maintain the constant position of material samples.

## Figures and Tables

**Table 1:** Geometrical properties of polymer–metal composites.

Nr Sample	Geometric Dimensions [mm]	Weight [g]	Surface Area [mm${}^{2}$]
1	30.5 × 4.4 × 0.3	0.1	134.2
2	44 × 8 × 0.3	0.22	320

## Data Availability

Not applicable.
